# Pedotransfer Functions for Estimating Soil Bulk Density Using Image Analysis of Soil Structure

**DOI:** 10.3390/s23041852

**Published:** 2023-02-07

**Authors:** Maja Bryk, Beata Kołodziej

**Affiliations:** Institute of Soil Science and Environment Management, University of Life Sciences in Lublin, Leszczyńskiego 7, 20-069 Lublin, Poland

**Keywords:** bulk density, image analysis, model, pedotransfer functions

## Abstract

Soil bulk density is one of the most important soil properties. When bulk density cannot be measured by direct laboratory methods, prediction methods are used, e.g., pedotransfer functions (PTFs). However, existing PTFs have not yet incorporated information on soil structure although it determines soil bulk density. We aimed therefore at development of new PTFs for predicting soil bulk density using data on soil macrostructure obtained from image analysis. In the laboratory soil bulk density (*BD*), texture and total organic carbon were measured. On the basis of image analysis, soil macroporosity was evaluated to calculate bulk density by image analysis (*BDim*) and number of macropore cross-sections of diameter ≥5 mm was determined and classified (*MP5*). Then, we created PTFs that involve soil structure parameters, in the form *BD*~*BDim* + *MP5* or *BD*~*BDim*. We also compared the proposed PTFs with selected existing ones. The proposed PTFs had mean prediction error from 0 to −0.02 Mg m^−3^, modelling efficiency of 0.17–0.39 and prediction coefficient of determination of 0.35–0.41. The proposed PTFs including *MP5* better predicted boundary *BD*s, although the intermediate *BD* values were more scattered than for the existing PTFs. The observed relationships indicated the usefulness of image analysis data for assessing soil bulk density which enabled to develop new PTFs. The proposed models allow to obtain the bulk density when only images of the soil structure are available, without any other data.

## 1. Introduction

Soil bulk density is one of the most important soil properties. On the one hand, it describes the quality of the soil since soil bulk density directly reflects soil porosity and compaction [1,2,3] which influence water and air soil properties [4,5,6]. On the other hand, soil bulk density is a crucial parameter used in balancing and modelling of various processes in the environment. Therefore, it is necessary for knowledge-based soil management and engineering applications, such as smart agriculture, forestry, terrestrial ecosystem management and land reclamation [7,8,9,10,11]. Soil bulk density is vital for the estimation of carbon sequestration in a specific soil layer [12,13]. Moreover, it allows to convert the soil’s abundance of nutrients and water from a mass basis to a volume or area basis.

Soil bulk density can be determined in two ways: directly in laboratory using core samples by a thermogravimetric method and indirectly using prediction methods, for example [14,15,16,17,18,19]. Indirect methods include pedotransfer functions (PTFs) which are equations or algorithms representing relationships between soil properties different in difficulty of their measurement or their availability [20,21,22]. PTFs use thereby other parameters of soil to estimate its bulk density.

Many models have been developed to predict bulk density from physical and chemical soil data, for example [15,21,23,24,25]. In developing PTFs, soil texture (sand, silt and clay contents) and organic matter content were the most applied predictors [4,14,18,22,23,26]. However, existing pedotransfer functions have not yet incorporated information on soil structure which, by definition, determines soil bulk density. In this context, soil structure expressed by means of pore and solid phase element size and arrangement can potentially enhance PTFs [5,27]. One method for direct qualitative and quantitative assessment of soil structure is image analysis. It yields valuable numerical indices of structure which can be used to search for relationships between bulk density and structure enabling development of PTFs.

Hence, we aimed at development of new PTFs using data on soil macrostructure obtained from computer-aided image analysis. The objectives of this study were: (i) to test the relationships among selected soil parameters measured in the laboratory and structure parameters obtained from image analysis, (ii) to create PTFs for assessment of soil bulk density that involve soil structure parameters, (iii) to compare the proposed PTFs with selected existing ones and (iv) to validate the proposed and existing PTFs.

For this purpose, we measured in the laboratory: soil bulk density, particle density, texture and total organic carbon. On the basis of image analysis, soil macroporosity was evaluated and then used to calculate bulk density by image analysis. Number of macropore cross-sections of diameter ≥5 mm was also determined and classified. Several statistical parameters were calculated to assess the quality of the studied PTFs: mean error, standard deviation of the prediction error, root mean square error, modelling efficiency and coefficient of determination.

## 2. Materials and Methods

### 2.1. Data Sets

The data used in this study included soil structure images from the collection of the Institute of Soil Science, Environment Engineering and Management at the University of Life Sciences in Lublin. For the purposes of this research, a training and a validation set were prepared.

For the training set, we employed images representing horizons of an Arenosol (AR) and two Podzols (PZ) developed from sand and a Cambisol (CM), a Luvisol (LV) and a Phaeozem (PH) developed from loess. Eight images were used per each studied horizon except for three horizons for which there were five, six or seven images available. A total of 319 images of the structure were included in the training set. Due to the large differences in bulk densities of mineral and organic horizons of the soils studied, calculations were carried out in two ways: using the whole set of both organic and mineral horizons (set *o+m*) or on a subset including only mineral horizons (subset *m*). A subset of organic horizons was not identified owing to their small number.

For the validation set, we utilized images representing mineral horizons of Arenosols developed from sand, Luvisols developed from loam and loess and Phaeozems developed from loess. We used one image per each studied horizon thus obtaining in total 52 images of soil structure. The photographs of soil structures in the validation set with additional information on the soils can be found in [28].

### 2.2. Selected Physical and Chemical Properties

Soil texture was assessed by a combination of the hydrometer and the wet-sieve methods [29] to measure fractions of: sand 0.05–2 mm (*Sand*), silt 0.002–0.05 mm (*Silt*), and clay <0.002 mm (*Clay*) in g/100g. Texture classes (*TextureClass*) were coded as: 0 for organic horizons, 1 for sandy horizons (S), 2 for silty horizons (Si) and 3 for silt loam horizons (SiL). Then, geometric diameter of particles (*GDP*) and geometric standard deviation of particle size (*GSD*) [30] were calculated from the following equations:

(1) $$\mathrm{GDP}=\exp(a)\;\mathrm{where}\;a=\sum m_{i}\ln d_{i}$$

(2) $$\mathrm{GSD}=\exp(b)\;\mathrm{where}\;b=\sqrt{\sum m_{i}{\left(\ln d_{i}\right)}^{2}-a^{2}}$$

where: *m_i_*—content of fraction *i* (g g^−1^), *d_i_*—arithmetic mean of particle diameters of fraction *i*. The summation was taken over three fractions: sand, silt and clay with arithmetic means of the diameters of the fractions: 1.0025 mm, 0.026 mm and 0.001 mm, respectively.

*GDP* (Equation (1)) and *GDS* (Equation (2)) represent particular combination of sand, silt and clay basing on the assumption that particle size distribution in soil is approximately log-normal [30]. *GDP* and *GDS* allow moreover for the quantitative representation of soil texture, which was useful in searching for correlations with other soil parameters.

Particle density (*PD1*, Mg m^−3^) was measured by the pycnometer method [31]. Total organic carbon (*TOC*, g/100 g) was estimated by wet oxidation with dichromate (VI) in sulphuric (VI) acid [32]. Organic matter content (*OM*, g g^−1^) was calculated by multiplying the *TOC* value by 1.724.

Characteristics of soil samples included in the training set are presented in Table A1. The studied horizons had texture of sand (S), silt loam (SiL) and silt (Si). *GDP* represented the mean particle size and ranged from 0.020 for fine-textured soils to 0.984 for coarse-textured ones. *GSD* was between 1.294 and 9.620 for soils with a narrow and wide particle size distribution, respectively. *PD1* was in the range 1.48–2.73 Mg m^−3^. *TOC* in the organic horizons was between 14.88 and 31.68 g/100 g while for the mineral horizons 0.04–3.04 g/100 g.

Characteristics of soil samples included in the validation set are presented in Table A2. The studied horizons had texture of sand (S), loamy sand (LS), sandy loam (SL) and silt loam (SiL). *PD*s were not available, therefore literature data were used [33]. Generally, *PD* of 2.65 Mg m^−3^ was applied. In the few cases of soils with a relatively high *TOC* or a high proportion of organic material visible in the structure images, *PD* equalling 2.00 Mg m^−3^ was used. *BD* for the training set was in the range 0.83–1.77 Mg m^−3^ while *BDim* calculated from image analysis data was 1.66–2.59 Mg m^−3^. *TOC* was between 0.02 and 2.53 g/100 g.

### 2.3. Bulk Density

For soil bulk density measurements, six soil samples with a preserved structure were collected vertically in metal cylinders (cores) per each soil horizon of the training and validation sets. The bulk density (*BD*, Mg m^−3^) was determined with the thermogravimetric method, on the basis of the ratio of the mass of soil dried at 105 °C to the initial volume of the soil (100 cm^3^). In the following development of PTFs, their recalibration and validation, the mean *BD* value was applied.

Bulk density was also assessed from image analysis data. The images of soil structures were obtained on the basis of resin-impregnated soil blocks prepared according to the methodology described earlier [28,34,35]. Briefly, the polished faces of soil blocks were scanned in colour at a 1200 dpi resolution and then processed with the image analysis program Aphelion (Adcis SA) to obtain binary (black & white) images of ca. 8 × 9 cm. The used scanning resolution limited the minimum diameter of the measured pore cross-section to 21.17 µm. Utilizing the data from the entire extent of each image, the areas of pore cross-sections were measured and then macroporosity (*AA*, m^2^ m^−2^), was calculated as the relation of cross-section of pores to the area of the image. Subsequently, for each horizon in the training set, the mean *AA* was computed and this value was used in further calculations of PTFs.

Moreover, one image per each horizon of the validation set was used to obtain the *AA* value. Finally, using the relationship for calculating soil total porosity based on bulk and particle densities, bulk density by image analysis (*BDim*, Mg m^−3^), was determined as:

*BDim* = *PD* (1 − *AA*), (3)

where: *PD* is the particle density (Mg m^−3^) and *AA* is the macroporosity by image analysis (m^2^ m^−2^).

Two different values of particle density were taken for each sample (i.e., soil horizon): one measured in the laboratory (*PD1*) and the other based on soil science literature (*PD2*) [33] and chosen with relevance to characteristics of a specific soil horizon. This approach was adopted due to the fact that the exact value of soil particle density may not be known. Furthermore, we wanted to test whether and how much the particle density value influences the modelling performance. Consequently, two bulk densities by image analysis were obtained, *BDim1* and *BDim2* (Table A1 and Table A2).

### 2.4. Macropores

**Figure 1:**
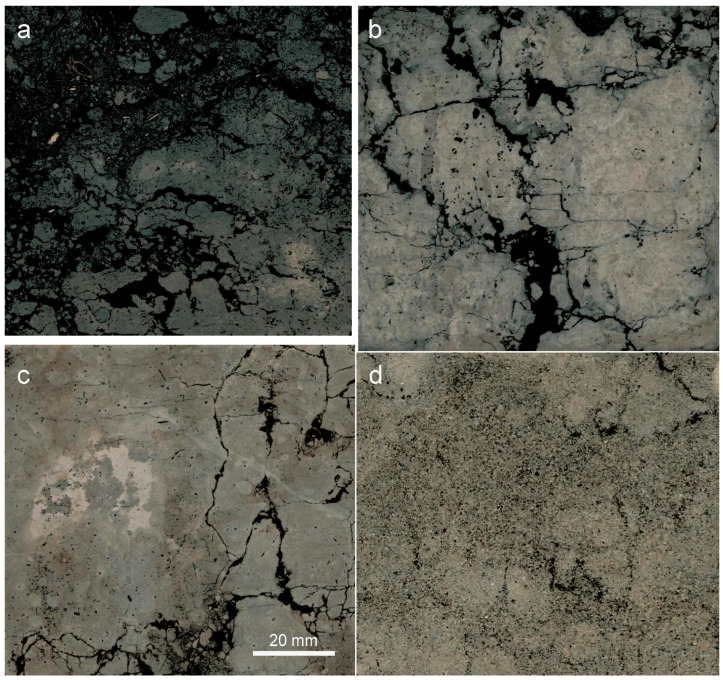
Representative images of soil macrostructure to demonstrate the *MP5* classes; pores are marked with black. (**a**) CM02.A, *MP5* = 1, (**b**) LV04.E, *MP5* = 2, (**c**) PH05.C, *MP5* = 3, (**d**) PZ07.C, *MP5* = 4.

**Figure 2:**
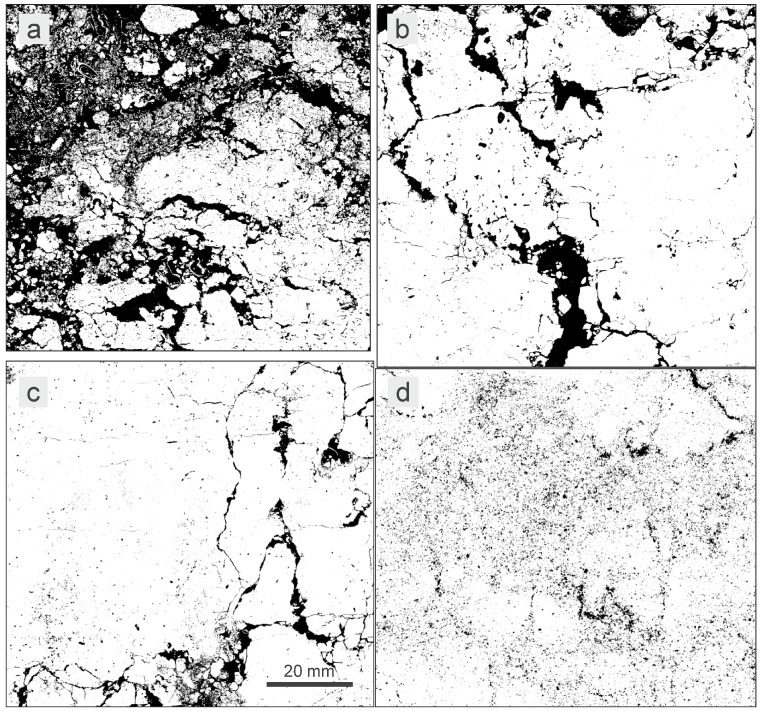
Binary images of soil macrostructure corresponding to the images shown in Figure 1; pores are marked with black. (**a**) CM02.A, (**b**) LV04.E, (**c**) PH05.C, (**d**) PZ07.C.

Images of soil structure were used to assess the number of macropore cross-sections with diameters ≥5 mm. This characteristic was expressed by the *MP5* parameter for which small values were adopted for lower bulk density and large values for higher bulk density. This corresponded to the general observation that with higher volume of macropores, lower soil bulk density should be expected. Consequently, the *MP5* values were as follows: 1 when the number of macropores was over 10, 2 for 6–10 macropores, 3 for 1–5 macropores and 4 when the number of macropores was 0 (Table A1 and Table A2, Figure 1 and Figure 2). The parameter basing on the identification of macropore cross-sections was chosen to utilize the unique information contained in the structure images—such data cannot be obtained using indirect methods of soil structure assessment like water or air permeability measurement. It is also of practical importance that the large macropores are easy to detect in the images of soil structure.

### 2.5. Modelling and Recalibration of Pedotransfer Functions

**Figure 3:**
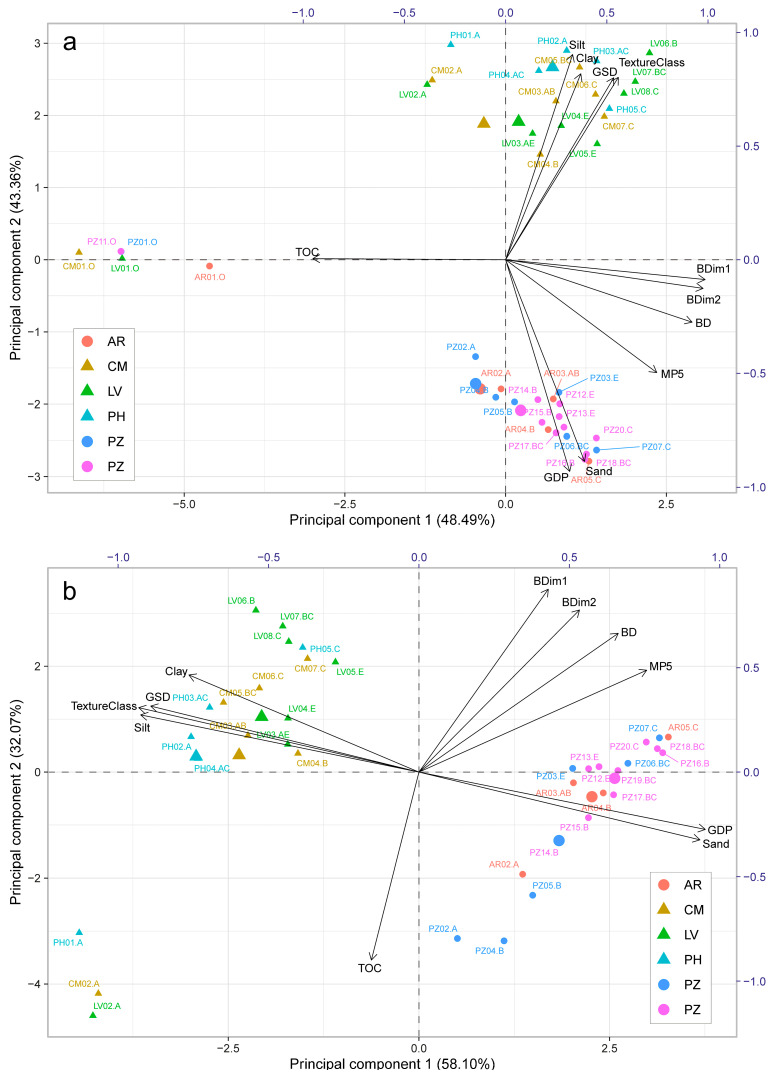
Principal component analysis (PCA) for soil samples included in the training set. (**a**) Biplot for both organic and mineral horizons, (**b**) biplot for mineral horizons. Biplot features: soil horizons—bottom and left axis showing PC1 and PC2 score, respectively; parameters—top and right axis showing loadings on PC1 and PC2, respectively.

First, we aimed at finding general relations among parameters in the training data set using the principal component analysis (PCA, Figure 3). For all soil horizons tested (Figure 3a), as well as for the subset of mineral horizons (Figure 3b), the soil bulk density calculated from the image analysis and that measured in the core samples were found to be positively correlated. It provided the basis for further calculations. Moreover, a strong positive correlation was observed between the bulk densities and the *MP5* parameter. Analysing the PCA graphs, we also confirmed that the bulk densities were negatively and strongly correlated with *TOC*. However, no strong relationships of bulk densities with texture-related parameters: *TextureClass*, *Sand*, *Silt*, *Clay*, *GDP*, *GSD* were found.

Considering the above, the following parameters were employed in the PTFs in this study: *BDim*, *MP5* and *TOC*. Consequently, we proposed the following forms of the relations between parameters derived from soil structure images and bulk density:

*BD* = A1 · *BDim1* + B1 · *MP5* + C1, (4)

*BD* = A2 · *BDim2* + B2 · *MP5* + C2, (5)

*BD* = D1 · *BDim1* + E1, (6)

and *BD* = D2 · *BDim2* + E2, (7)

where: A1, A2, …, E1 and E2 are coefficients to be found using the training set.

The calculations of the coefficients were performed with the Solver tool of Microsoft Excel by minimising the sum of squares of the differences between bulk densities predicted with Equations (4)–(7) and bulk densities measured by the core method. The fitting was made on the training set utilizing the set *o+m* or the subset *m*. Finally, eight variants of the new PTFs (M01–M08) were developed as part of this research.

Next, given the aforementioned strong relationship between bulk density and *TOC*, we selected the following existing PTFs of high efficiency [26]:

*BD* = 1.660 − 0.318 *TOC*^0.5^ (8)

*BD* = 0.159 × 1.561/[1.561 *OM* + 0.159 (1 − *OM*)] (9)

*BD* = 0.167 × 1.526/[1.526 *OM* + 0.167 (1 − *OM*)] (10)

where: *TOC*—total organic carbon content (g/100 g), *OM*—organic matter content (g g^−1^).

The three models presented in the Equations (8)–(10) [36,37,38], are later referred to as M09, M14 and M15, respectively. The PTFs were then recalibrated on the training set using the set *o+m* or the subset *m* with the Solver tool of Microsoft Excel as described above. The applied procedure resulted in six modified PTFs: M10–M13, M16 and M17. In M10 and M12, the constant term was set equal to the maximum bulk density of the training set, which was 1.747.

### 2.6. Statistical Tests for Model Evaluation

The performance of all 17 PTFs studied was checked on the training and the validation sets. The same parameters were adopted to evaluate all models, in order to facilitate a variety of comparisons. The following characteristics of the PTFs were calculated: ME—mean error, SDE—standard deviation of the prediction error, RMSE—root mean square error, EF—modelling efficiency, R^2^—coefficient of determination:

(11) $$\mathrm{ME}=\frac{1}{N}\sum_{1}^{N}\left(BD_{pi}-BD_{i}\right)$$

(12) $$\mathrm{SDE}=\sqrt{\frac{1}{N-1}\sum_{1}^{N}{\left(\left(BD_{pi}-BD_{i}\right)-\mathrm{ME}\right)}^{2}}$$

(13) $$\mathrm{RMSE}=\sqrt{\frac{1}{N}\sum_{1}^{N}{\left(BD_{pi}-BD_{i}\right)}^{2}}$$

(14) $$\mathrm{EF}=1-\frac{\sum_{1}^{N}{\left(BD_{pi}-BD_{i}\right)}^{2}}{\sum_{1}^{N}{\left(BD_{i}-\bar{BD}\right)}^{2}}$$

(15) $$R^{2}=\frac{{\left[\mathrm{cov}\;\left(BD_{i},BD_{pi}\right)\right]}^{2}}{\mathrm{var}\;\left(BD_{i}\right)\cdot \mathrm{var}\;\left(BD_{pi}\right)},$$

where *BD_pi_* and *BD_i_* are the predicted and measured soil bulk densities, respectively, $\bar{BD}$ is the arithmetic mean of the measured *BD* values while *N* is the total number of observations equalling 42 for the training set (including 37 mineral horizons) and 52 for the validation set.

The ME (Equation (11)) reveals a positive or negative bias of a PTF, indicating an average tendency for overestimation or underestimation, respectively. The SDE (Equation (12)) shows the random variation of the predictions after correction for the global bias. The RMSE (Equation (13)) is a measure of the overall error of the prediction. For best performing models, the ME, SDE and RMSE should be as small as possible. The EF, the modelling efficiency (Equation (14)) [39], on the other hand, should be as high as possible. The coefficient of determination R^2^ (Equation (15)) is a measure of the strength of the linear relationship between measurements and predictions and indicates the fraction of the variation that is shared between them [24].

Plots showing predicted versus measured bulk density values with reference to the 1:1 line were also prepared for visual assessment of the fit quality.

Statistical calculations were performed in Microsoft Excel.

## 3. Results and Discussion

### 3.1. Bulk Density

**Figure 4:**
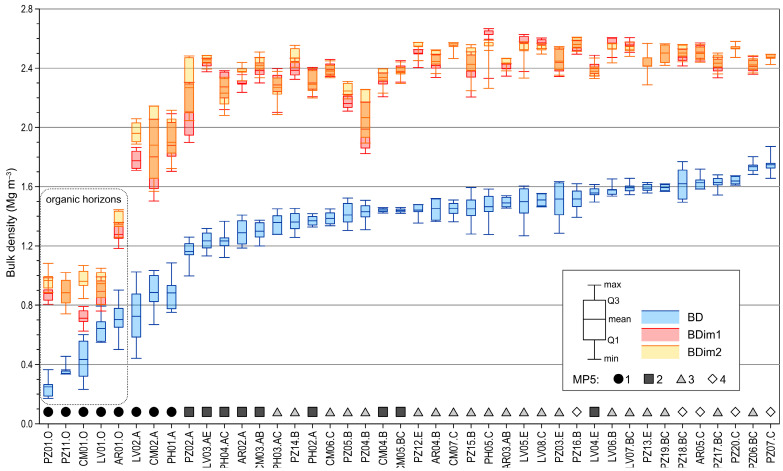
Distribution of bulk density values for each horizon in the training set. *BD*—bulk density measured using core samples, *BDim1* and *BDim2*—bulk density by image analysis calculated with *PD1* or *PD2*, respectively.

The bulk density measured using the soil cores (*BD*) and the bulk density calculated from the image analysis (*BDim1* and *BDim2*) are presented in Figure 4. *BD* ranged from 0.249 to 1.747 Mg m^−3^. The bulk densities by image analysis were always higher than the bulk density measured in the core samples. Namely, *BDim1* ranged from 0.711 to 2.596 Mg m^−3^ and *BDim2* from 0.833 to 2.560 Mg m^−3^. Organic horizons had the lowest *BD*, *BDim1* and *BDim2*. The bulk densities by image analysis showed a similar trend to the bulk density by core samples. In addition, the classes of quantity of pores ≥5 mm (*MP5*) were marked in the graph. As expected, the samples with the highest content of large pores had the lowest bulk densities.

### 3.2. Proposed Pedotransfer Functions

**Figure 5:**
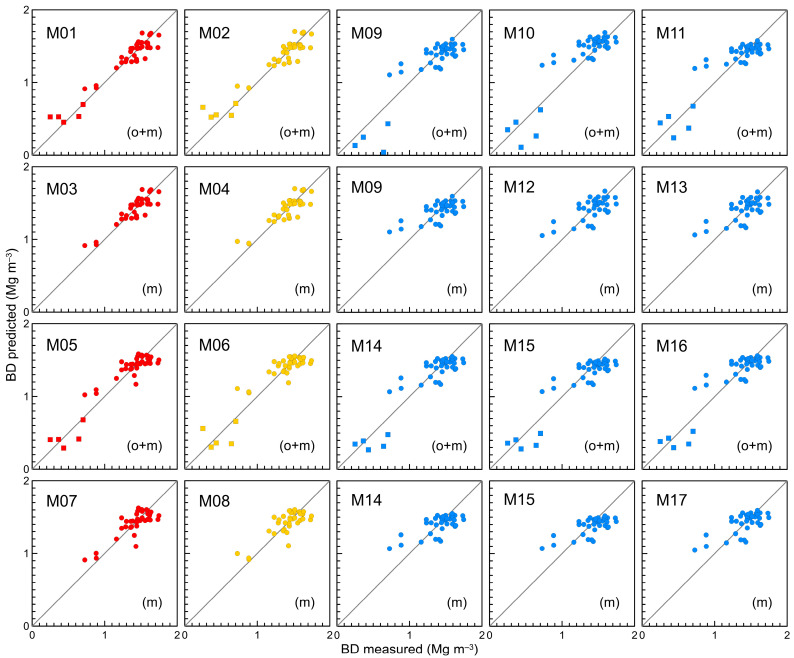
Measured vs. predicted bulk density by the M01–M17 pedotransfer functions with reference to the 1:1 line (training set). Circles—mineral horizons, squares—organic horizons. M01–M08: yellow and red symbols—pedotransfer functions developed with *BDim1* and *BDim2*, respectively.

The PTFs developed in the study are shown in Table 1 and Figure 5. Results of statistical evaluation of the PTFs showed a positive or zero ME for all models. Generally, for the proposed PTFs, the ME values were low, ranging from 0.00 to 0.01 Mg m^−3^. Root mean square errors were in the range from 0.10 to 0.15 Mg m^−3^. The RMSE was lower, i.e., more favourable, for all models including *MP5*, compared to analogous models without *MP5*, see for example M01 and M05. The low ME and RMSE values resulted in high coefficients of determination for the studied functions, R^2^ = 0.64–0.92.

The modelling efficiency (EF) was equal or greater than 0.64 and reached a maximum of 0.92. When comparing the analogous models, for example M01 and M05, we found that including *MP5* in the equation increases EF. Furthermore, higher EFs were achieved for functions based on *BDim1* compared to those including *BDim2*. The process of fitting function coefficients during the modelling of PTFs yielded higher EFs when conducted on the set *o+m*, as can be seen by comparing, for example, M01 and M03.

The analysis of Figure 5 confirmed the above observations regarding the statistical indices describing the proposed models. For M01, the data points were situated closer to the 1:1 line in comparison to M05 and M07. Using the particle density value obtained from the literature to calculate the bulk density by image analysis deteriorated this fit slightly, as could be seen while comparing the pairs M01 and M02, M03 and M04, M05 and M06, M07 and M08. The comparison of the M01 and M05 plots revealed that the inclusion of the *MP5* parameter in the model increased or decreased the predicted bulk density. As a result, the location of data points had changed and they moved closer to the 1:1 line.

In conclusion, we stated that the statistical indices obtained for the proposed PTFs were favourable, indicating the possible usefulness of image analysis data for assessing soil bulk density. Models M01 and M02 were the best, which suggested that a training set with a wide range of the *BD* values (containing in particular organic horizons with relatively low *BD*s) proved to be preferable during the model development. The inclusion of a parameter related to soil structure, in this study represented by the number of macropores, also had a positive effect on the modelling efficiency.

### 3.3. Existing and Recalibrated Pedotransfer Functions

We tested three existing PTFs models to calculate bulk density based on organic carbon or organic matter content, following Abdelbaki’s recommendation [26]. He evaluated forty-eight PTFs that required particle size distribution, organic matter and organic carbon contents and stated that the functions depending on the organic matter or organic carbon as inputs were characterised by the best performances. What is more, the relationship between soil bulk density and organic matter has been confirmed in many studies, therefore PTFs based on these parameters are commonly used in environmental research [15,40]. It is also well established in soil science that the general trend of the linear correlation between the organic matter or organic carbon content and the bulk density is negative: with the increase in the amount of organic matter in the soil, bulk density decreases, for example [41,42,43,44].

The first model (M09) was formulated by Manrique and Jones [36] using data of soil pedons from the continental USA, Hawaii, Puerto Rico and some foreign countries, with soils grouped into subsets according to their Soil Taxonomy classification. The model created had a simple form and showed a decrease in bulk density as *TOC* increased [44,45].

To enhance the fit of the original model M09 to local data, we recalibrated the model on the training set using both the set *o+m* and the subset *m*. As stated above, the recalibration of M09 was performed with the constant term equal to the maximum bulk density of the training set or without this restriction. The procedure yielded models M10 and M12, and M11 and M13, respectively. Results of statistical evaluation of the models M09–M13 are presented in Table 2 and their graphical representations are shown in Figure 5.

The original model M09 tested on the set *o+m* underestimated *BD*, because ME equalled −0.06 Mg m^−3^. M09 had six times the absolute value of ME compared to our best models M01 and M02. SDE and RMSE were almost two times higher than for M01 or M02.

Another indicator of the model quality is EF. In the study, EF greater than or equal 0.20 and less than 0.50 indicated medium while EF greater or equal to 0.50—high performance of a model [26]. Following the criteria, M09 had a high efficiency with EF of 0.69.

Models M10 and M11, with parameters of M09 tailored to the set *o+m*, as expected, performed better than M09 in terms of ME, SDE, RMSE and EF. For M10 we observed, however, a relatively high overestimation of *BD*, 0.06 Mg m^−3^. Recalibration led to an increase in EF that for M11 reached 0.78. Despite the improvement of the statistical indices against M09, the models M10 and M11 did not reach such favourable values as for M01 and M02.

Next, the model M09 was tested and recalibrated using the subset *m*. Similarly, as with the set *o+m*, *BD* was underestimated, although the absolute value of ME was lower and equalled −0.02 Mg m^−3^. Despite a decrease of SDE and RMSE, there was no improvement in EF and lower R^2^ was observed.

After recalibration, models M12 and M13 performed better than M09 (m) in terms of ME, SDE, RMSE and EF, similarly as described above for testing with the set *o+m*. Moreover, M12 and M13 showed lower values of ME, 0.00 Mg m^−3^, in comparison to this parameter of M01 and M02, 0.01 Mg m^−3^. On the other hand, SDE, RMSE, EF and R^2^ were considerably less favourable than for M01 and M02 and consequently EF was classified as medium.

The second and third model considered in the study, M14 and M15, were proposed by Prévost [37] and Han et al. [38], respectively. Prévost [37] developed the formula based on the two sandy till soils in northern Québec taking into account bulk density for both organic and mineral soil samples. Han et al. [38] recalculated the Prévost’s model for estimating bulk density of soils in China.

Following the previous procedure for M09, the original models M14 and M15 were tested on the training set using both the set *o+m* and the subset *m*. Then, using the same data sets, we recalibrated both models to obtain models M16 and M17. Results for the models M14–M17 are presented in Table 2 and Figure 5.

The existing models M14 and M15 verified on the set *o+m* underestimated *BD* and ME was −0.03 Mg m^−3^ and −0.05 Mg m^−3^, respectively. Compared to M01 and M02, the absolute values of the measured statistical indices were higher, i.e., less favourable, for M14 and M15. The efficiency of both models was high, 0.81, nevertheless it was lower than for M01 and M02.

The M16 model resulting from recalibration using the set *o+m* was characterised by a zero ME. Nonetheless, other parameters such as SDE, RMSE and R^2^ were virtually identical to those of M14 and M15. EF was the highest (0.81) among the three models. M16 performed almost as well as M01 and M02, as evidenced by favourable values of ME and only slightly lower EF and R^2^.

The models M14 and M15 were subsequently tested and recalibrated using the subset *m*. Similarly, as with the set *o+m*, *BD* was underestimated, however to a lesser extent, since ME reached the values of −0.02 Mg m^−3^ and −0.04 Mg m^−3^ for M14 and M15, respectively. SDE and RMSE decreased (i.e., improved) slightly while EF and R^2^ decreased (i.e., deteriorated) considerably.

The model M17 resulting from recalibration revealed zero ME. SDE, RMSE and R^2^ decreased slightly while EF increased compared with M14 and M15 tested on the subset *m*, which suggested the improvement of model quality. For M17, however, the EF and R^2^ values were only half that of models M01 and M02, indicating lower performance of the model M17.

Figure 5 presents scatter plots for the models M09–M17. One result of the negative bulk density for M09 was not shown, since it was not physically correct. The distribution of data points in the plots confirmed the earlier analysis of the statistical indices for these models (Table 2). Recalibrating M09 based on the set *o+m* to the models M10 and M11 resulted in a more realistic bulk density without negative values. Of the models M09, M10 and M11, the latter yielded the data points which were the most concentrated around the 1:1 line. The models M09, M12 and M13 tested on the subset *m* produced similarly arranged data points, but for M12 and M13 an increase in the range of *BD* predicted and consequently a slight expansion of the data-point cloud was observed. The models M14–M16 tested on the set *o+m* and M14, M15 and M17 tested on the subset *m* resulted in equivalent predictions of *BD*.

Comparing M09–M17 with the models M01–M08, we observed that M09–M17 were characterised by a bias of the *BD* predicted against *BD* measured, consisting in an overestimation of the bulk density for low values and an underestimation for high values. Moreover, the main axis of the observed data-point cloud rotated clockwise with respect to the 1:1 line. This behaviour is characteristic of the TOC-based PTFs, as confirmed by previous studies, for example [23,26,42].

### 3.4. Validation of Proposed, Existing and Recalibrated Pedotransfer Functions

**Figure 6:**
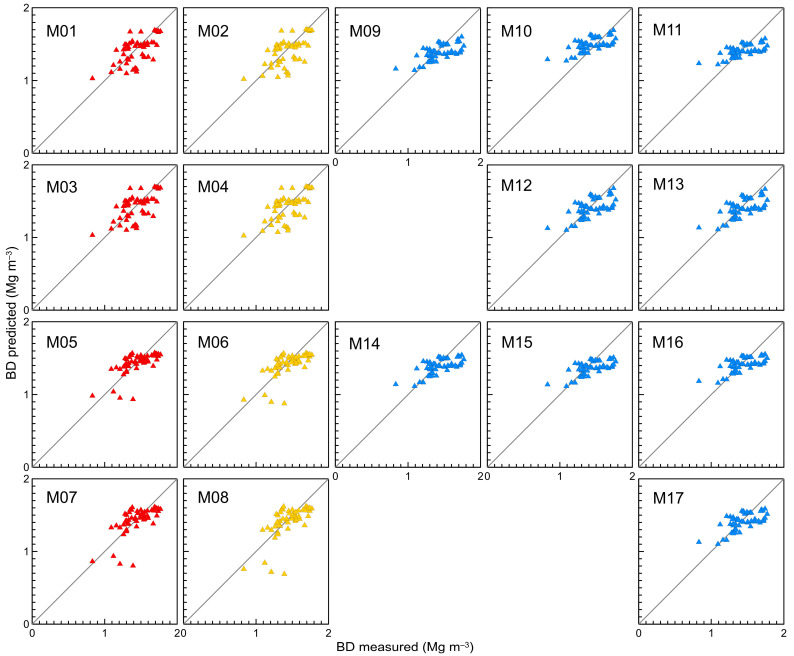
Measured vs. predicted bulk density by the M01–M17 pedotransfer functions with reference to the 1:1 line (validation set). M01–M08: yellow and red symbols—pedotransfer functions developed with *BDim1* and *BDim2*, respectively.

The results for all studied models are listed in Table 3 with ME, SDE, RMSE, EF and R^2^ values. The relationships of measured versus predicted bulk density with reference to the 1:1 line are shown in Figure 6.

The results revealed that the models M03, M05, M07 had zero ME while M10 had ME of 0.07 Mg m^−3^. The other models had negative ME, from −0.01 Mg m^−3^ to −0.05 Mg m^−3^. Thus, most models underestimated *BD*, except for M10 which yielded overestimated *BD* values. ME values could therefore be characterised as quite diverse. Narrower ranges were recorded for SDE and RMSE, 0.15–0.18 Mg m^−3^. Efficiency (EF) was in the range 0.17–0.41. Most models achieved medium performance with EF equal or greater than 0.20. It was noted that R^2^ was in the range 0.35–0.42.

Figure 6 presents scatter plots for the models M01–M17 tested using the validation set. Analysing the data-point distributions in the graphs, it was found that the models M12 and M13 yielded very similar results with data points located closest to the 1:1 line. Compared to M12 and M13, the models M09–M11 and M14–M17 generated more clustered data points, but again overestimation of bulk density was evident for lower values and at the same time underestimation for higher ones. The best *BD* predictions were achieved for bulk densities representing intermediate values in the data set.

Proposed models M01–M04 performed not as good as expected if we consider the statistical parameters achieved (Table 3). However, the graphs showed some advantage of these models over M12 and M13, as the proposed models M01–M04 better predicted boundary bulk density values in the validation set. The models M01–M04 were characterised by a greater scattering of the data points in the middle range of the *BD* measured in comparison to M12 and M13.

The M05–M08 plots showed a dense data-point clouds with distinct four points for which the predicted *BD* was equal to or less than one. In the graphs of M07 and M08, the main groups of data points were located closer to the 1:1 line than for M05 and M06.

## 4. Summary and Conclusions

Soil bulk density calculated from the image analysis and that measured in the core samples were found to be positively correlated. Moreover, a strong positive correlation was observed between the bulk density and the quantity of pores of diameter ≥5 mm detected in the images of soil structure. The observed relationships indicated the usefulness of image analysis data for assessing soil bulk density, which enabled to develop new PTFs.

The proposed PTFs were designed to depend on the bulk density by image analysis with or without parameter related to the number of macropores. The inclusion of that parameter, which could be obtained from images of soil structure, had a beneficial effect on the modelling efficiency.

Sets of samples with a wide range of bulk density values, containing in particular organic horizons with relatively low bulk densities, proved to be better during the model development and validation. The proposed and existing models in which the coefficients of the equations were fitted using the training set with both organic and mineral horizons showed the highest performances, indicated by the highest efficiencies and coefficients of determination. The analogue models with the coefficients fitted using the subset of mineral horizons or the models tested on the validation set (consisting only of mineral horizons) showed reduced performances with lower efficiencies and coefficients of determination.

In the study we confirmed that the bulk density was negatively and strongly correlated with organic carbon content. Therefore, we selected for analysis three existing PTFs which included organic carbon or organic matter content. Recalibration of the existing models using the training set increased their performance, evidenced by an increase of the modelling efficiency.

The proposed models tested on the training set performed better than the existing and recalibrated models, when comparing separately the models tested on the set of both organic and mineral horizons and on the subset of mineral horizons.

Considering all models tested on the validation set, it was found that the proposed models were similar to the existing and recalibrated models in terms of the modelling efficiency.

The proposed models including only bulk density by image analysis with coefficients fitted with the training set of both organic and mineral horizons were characterised by the modelling efficiencies similar to those of the existing models while achieving a lower mean error. The proposed models including bulk density by image analysis and the parameter related to the macropore number performed not as good as the existing and recalibrated models. However, the proposed models better predicted boundary bulk density, i.e., they neither overestimated bulk density for lower values nor underestimated it for higher ones.

The proposed models allow bulk density to be obtained when only images of soil structure are available, without any other data. Furthermore, macroporosity (a fraction of the area occupied by one phase) and macropore number used in our research to create the pedotransfer functions can be easily measured using open-source image processing software. This provides the opportunity to supplement the databases with missing bulk density values. The proposed models also indicate the need to include structure parameters in the studies of bulk density, as it depends on soil’s structure. The developed pedotransfer functions linked for the first time soil bulk density with structural characteristics assessed by a direct visualization method. They build therefore a valuable foundation for further research.

## Figures and Tables

**Table 1:** Performance of proposed pedotransfer functions for the training set.

Model	Sample Set	Equation	ME Mg m^−3^	SDE Mg m^−3^	RMSE Mg m^−3^	EF	R^2^
M01	*o+m* *	*BD* = 0.432 *BDim1* + 0.146 *MP5*	0.01	0.11	0.11	0.92	0.92
M02	*o+m*	*BD* = 0.398 *BDim2* + 0.171 *MP5*	0.01	0.13	0.12	0.89	0.90
M03	*m*	*BD* = 0.434 *BDim1* + 0.146 *MP5*	0.00	0.10	0.10	0.79	0.79
M04	*m*	*BD* = 0.415 *BDim2* + 0.159 *MP5*	0.00	0.11	0.11	0.76	0.76
M05	*o+m*	*BD* = 0.688 *BDim1* − 0.199	0.00	0.13	0.13	0.87	0.87
M06	*o+m*	*BD* = 0.748 *BDim2* − 0.357	0.00	0.15	0.15	0.84	0.84
M07	*m*	*BD* = 0.871 *BDim1* − 0.635	0.00	0.13	0.13	0.67	0.67
M08	*m*	*BD* = 1.007 *BDim2* − 0.976	0.00	0.13	0.13	0.64	0.64

* *o+m*—set of both organic and mineral horizons, *m*—subset of mineral horizons, *BD*—bulk density, *BDim1* and *BDim2*—bulk density by image analysis calculated with *PD1* or *PD2*, respectively, *MP5*—class of number of pore cross-sections of diameter ≥5 mm, ME—mean prediction error, SDE—standard deviation of the prediction error, RMSE—root mean square prediction error, EF—modelling efficiency, R^2^—prediction coefficient of determination.

**Table 2:** Performance of existing and recalibrated pedotransfer functions for the training set.

Model	Sample Set	Equation	ME Mg m^−3^	SDE Mg m^−3^	RMSE Mg m^−3^	EF	R^2^
M09 [36]	*o+m* *	*BD* = 1.660 − 0.318 *TOC*^0.5^	−0.06	0.20	0.21	0.69	0.78
M10	*o+m*	*BD* = 1.747 − 0.291 *TOC*^0.5^	0.06	0.19	0.19	0.73	0.78
M11	*o+m*	*BD* = 1.625 − 0.246 *TOC*^0.5^	0.00	0.18	0.17	0.78	0.78
M09 [36]	*m*	*BD* = 1.660 − 0.318 *TOC*^0.5^	−0.02	0.17	0.17	0.43	0.45
M12	*m*	*BD* = 1.747 − 0.397 *TOC*^0.5^	0.00	0.16	0.16	0.45	0.45
M13	*m*	*BD* = 1.734 − 0.384 *TOC*^0.5^	0.00	0.16	0.16	0.45	0.45
M14 [37]	*o+m*	*BD* = 0.159 × 1.561/[1.561 *OM* + 0.159 (1 − *OM*)]	−0.03	0.17	0.17	0.80	0.81
M15 [38]	*o+m*	*BD* = 0.167 × 1.526/[1.526 *OM* + 0.167 (1 − *OM*)]	−0.05	0.17	0.17	0.79	0.81
M16	*o+m*	*BD* = 0.178 × 1.572/[1.572 *OM* + 0.178 (1 − *OM*)]	0.00	0.17	0.16	0.81	0.81
M14 [37]	*m*	*BD* = 0.159 × 1.561/[1.561 *OM* + 0.159 (1 − *OM*)]	−0.02	0.16	0.16	0.46	0.48
M15 [38]	*m*	*BD* = 0.167 × 1.526/[1.526 *OM* + 0.167 (1 − *OM*)]	−0.04	0.16	0.17	0.42	0.48
M17	*m*	*BD* = 0.144 × 1.605/[1.605 *OM* + 0.144 (1 − *OM*)]	0.00	0.16	0.16	0.47	0.47

* *o+m*—set of both organic and mineral horizons, *m*—subset of mineral horizons, *BD*—bulk density, *BDim1* and *BDim2*—bulk density by image analysis calculated with *PD1* or *PD2*, respectively, *MP5*—class of number of pore cross-sections of diameter ≥5 mm, ME—mean prediction error, SDE—standard deviation of the prediction error, RMSE—root mean square prediction error, EF—modelling efficiency, R^2^—prediction coefficient of determination.

**Table 3:** Performance of the studied pedotransfer functions for the validation set.

Model	ME Mg m^−3^	SDE Mg m^−3^	RMSE Mg m^−3^	EF	R^2^
M01	−0.01	0.16	0.16	0.25	0.38
M02	−0.02	0.17	0.17	0.20	0.35
M03	0.00	0.16	0.16	0.30	0.38
M04	−0.01	0.17	0.17	0.25	0.36
M05	0.00	0.15	0.15	0.39	0.41
M06	−0.01	0.15	0.15	0.37	0.41
M07	0.00	0.16	0.16	0.30	0.41
M08	−0.02	0.18	0.18	0.17	0.41
M09	−0.04	0.15	0.15	0.36	0.41
M10	0.07	0.15	0.17	0.25	0.41
M11	−0.01	0.16	0.16	0.35	0.41
M12	−0.02	0.15	0.15	0.40	0.41
M13	−0.02	0.15	0.15	0.40	0.41
M14	−0.03	0.15	0.15	0.38	0.42
M15	−0.05	0.15	0.16	0.32	0.42
M16	−0.01	0.15	0.15	0.40	0.42
M17	−0.01	0.15	0.15	0.41	0.42

ME—mean prediction error, SDE—standard deviation of the prediction error, RMSE—root mean square prediction error, EF—modelling efficiency, R^2^—prediction coefficient of determination.

## Data Availability

The data presented in this study are available on request from the corresponding author.

## Appendix A

**Table A1:** Characteristics of the soil samples included in the training set.

Soil	Horizon	Symbol	*Sand* g/100 g	*Clay* g/100 g	*Silt* g/100 g	Texture	*GDP*	*GSD*	*PD1* Mg m^−3^	*PD2* Mg m^−3^	*BD* Mg m^−3^	*BDim1* Mg m^−3^	*BDim2* Mg m^−3^	*TOC* g/100 g	*MP5*
Arenosol	O	AR01.O	–	–	–	n.a. *	–	–	1.88	2.00	0.70	1.28	1.36	14.88	1
	A	AR02.A	95.0	0.0	5.0	S	0.835	2.217	2.56	2.65	1.29	2.30	2.38	1.49	2
	AB	AR03.AB	95.0	1.5	3.5	S	0.795	2.894	2.61	2.65	1.49	2.40	2.43	0.45	3
	B	AR04.B	99.0	0.5	0.5	S	0.951	1.734	2.62	2.65	1.45	2.42	2.45	0.47	3
	C	AR05.C	99.5	0.0	0.5	S	0.984	1.294	2.66	2.65	1.63	2.51	2.50	0.16	4
Cambisol	O	CM01.O	–	–	–	n.a.	–	–	1.48	2.00	0.43	0.71	0.96	31.68	1
	A	CM02.A	21.0	7.0	72.0	SiL	0.045	6.073	2.54	2.65	0.88	1.80	1.88	2.63	1
	AB	CM03.AB	18.0	10.0	72.0	SiL	0.036	6.240	2.61	2.65	1.30	2.38	2.42	0.63	2
	B	CM04.B	24.0	0.0	76.0	SiL	0.062	4.758	2.62	2.65	1.44	2.31	2.34	0.29	2
	BC	CM05.BC	17.0	18.0	65.0	SiL	0.027	7.720	2.66	2.65	1.44	2.38	2.37	0.29	2
	C	CM06.C	18.0	15.5	66.5	SiL	0.030	7.432	2.66	2.65	1.39	2.39	2.38	0.16	3
	C	CM07.C	13.0	12.0	75.0	SiL	0.028	5.653	2.65	2.65	1.45	2.55	2.55	0.35	3
Luvisol	O	LV01.O	–	–	–	n.a.	–	–	1.89	2.00	0.64	0.89	0.95	25.92	1
	A	LV02.A	29.0	6.0	65.0	SiL	0.062	6.951	2.40	2.65	0.73	1.77	1.96	3.04	1
	AE	LV03.AE	12.0	8.0	80.0	Si	0.031	4.735	2.63	2.65	1.23	2.44	2.46	0.64	2
	E	LV04.E	15.0	6.0	79.0	SiL	0.037	4.881	2.67	2.65	1.56	2.40	2.38	0.38	2
	E	LV05.E	15.0	6.0	79.0	SiL	0.037	4.881	2.70	2.65	1.50	2.57	2.52	0.16	3
	B	LV06.B	18.0	26.0	56.0	SiL	0.022	9.620	2.69	2.65	1.58	2.57	2.53	0.18	3
	BC	LV07.BC	17.0	20.0	63.0	SiL	0.025	8.127	2.68	2.65	1.59	2.56	2.53	0.24	3
	C	LV08.C	17.0	17.0	66.0	SiL	0.028	7.514	2.69	2.65	1.51	2.57	2.53	0.38	3
Phaeozem	A	PH01.A	14.0	15.0	71.0	SiL	0.027	6.423	2.62	2.65	0.88	1.88	1.90	1.59	1
	A	PH02.A	13.0	22.0	65.0	SiL	0.020	7.409	2.66	2.65	1.37	2.30	2.29	0.78	2
	AC	PH03.AC	19.0	22.0	59.0	SiL	0.025	9.085	2.67	2.65	1.36	2.29	2.27	0.34	3
	AC	PH04.AC	9.0	17.0	74.0	SiL	0.021	5.578	2.70	2.65	1.23	2.27	2.23	0.42	2
	C	PH05.C	12.0	14.0	74.0	SiL	0.026	5.794	2.73	2.65	1.46	2.60	2.52	0.23	3
Podzol	O	PZ01.O	–	–	–	n.a.	–	–	1.82	2.00	0.25	0.88	1.22	23.04	1
	A	PZ02.A	90.5	0.5	9.0	S	0.697	3.137	2.46	2.65	1.16	2.10	2.27	2.28	2
	E	PZ03.E	93.5	1.5	5.0	S	0.753	3.129	2.64	2.65	1.52	2.44	2.45	0.35	3
	B	PZ04.B	93.5	0.0	6.5	S	0.791	2.460	2.55	2.65	1.43	1.99	2.07	2.20	3
	B	PZ05.B	94.0	0.0	6.0	S	0.805	2.381	2.55	2.65	1.41	2.16	2.25	2.02	3
	BC	PZ06.BC	99.0	0.5	0.5	S	0.951	1.734	2.64	2.65	1.73	2.41	2.42	0.18	3
	C	PZ07.C	99.0	1.0	0.0	S	0.936	1.989	2.65	2.65	1.75	2.47	2.47	0.42	4
Podzol	O	PZ11.O	–	–	–	n.a.	–	–	2.00	2.00	0.36	0.88	0.88	19.68	1
	E	PZ12.E	95.0	1.0	4.0	S	0.808	2.670	2.60	2.65	1.44	2.50	2.55	0.62	3
	E	PZ13.E	97.0	1.0	2.0	S	0.870	2.342	2.65	2.65	1.59	2.41	2.41	0.04	3
	B	PZ14.B	94.0	0.5	5.5	S	0.792	2.605	2.56	2.65	1.36	2.39	2.47	2.00	3
	B	PZ15.B	97.0	0.0	3.0	S	0.898	1.865	2.60	2.65	1.45	2.38	2.43	1.07	3
	B	PZ16.B	99.5	0.0	0.5	S	0.984	1.294	2.63	2.65	1.52	2.54	2.56	0.71	4
	BC	PZ17.BC	98.0	0.0	2.0	S	0.932	1.667	2.62	2.65	1.63	2.41	2.43	0.89	3
	BC	PZ18.BC	98.5	0.0	1.5	S	0.949	1.559	2.62	2.65	1.62	2.47	2.50	0.45	4
	BC	PZ19.BC	97.0	0.0	3.0	S	0.898	1.865	2.65	2.65	1.59	2.50	2.50	0.72	3
	C	PZ20.C	98.0	1.5	0.5	S	0.887	2.402	2.65	2.65	1.64	2.53	2.53	0.83	4

* n.a.—not applicable, *GDP*—geometric diameter of particles, *GSD*—geometric standard deviation of particle size, S—sand, SiL—silt loam, Si—silt, *PD1*—particle density measured in the laboratory, *PD2*—standard particle density [33], *BD*—bulk density measured by core method, *BDim1* and *BDim2*—bulk density by image analysis calculated with *PD1* or *PD2*, respectively, *TOC*—total organic carbon content, *MP5*—class of number of macropore cross-sections of diameter ≥5 mm.

**Table A2:** Characteristics of soil samples included in the validation set.

Soil	Horizon	Texture	*PD*, Mg m^−3^	*BD*, Mg m^−3^	*BDim*, Mg m^−3^	*TOC*, g/100 g	*MP5*	Figure No. in [28]
Arenosol	A	S *	2.00	1.12	1.82	0.92	3	1
	B	S	2.65	1.46	2.49	0.29	3	2
Arenosol	A	S	2.65	1.52	2.50	0.24	2	24
	B	S	2.65	1.69	2.59	0.06	4	25
Arenosol	A	S	2.65	1.35	2.55	0.58	4	47
	B	S	2.65	1.71	2.57	0.12	4	48
Arenosol	A	S	2.65	1.42	2.42	0.72	3	63
	B	S	2.65	1.55	2.51	0.23	3	64
Arenosol	A	S	2.65	1.65	2.54	0.70	3	65
	A	S	2.65	1.72	2.56	0.60	4	66
Luvisol	A	SL	2.65	1.09	2.27	2.53	1	11
	E	SL	2.65	1.55	2.45	0.71	3	12
Luvisol	A	SiL	2.65	1.33	2.32	0.87	2	26
	E	SiL	2.65	1.37	2.55	0.59	3	27
	B	SiL	2.65	1.44	2.37	0.15	1	28
Luvisol	A	SL	2.65	1.50	2.46	0.80	3	29
	E	SL	2.65	1.74	2.53	0.02	4	30
Luvisol	A	SL	2.65	1.59	2.53	0.74	3	34
Luvisol	A	SiL	2.65	1.50	2.54	0.75	4	49
	E	SiL	2.65	1.54	2.43	0.22	2	50
Luvisol	A	SL	2.65	1.72	2.46	0.70	3	51
Luvisol	A	SiL	2.65	1.26	2.16	0.90	3	53
	E	SiL	2.65	1.60	2.42	0.58	2	54
Luvisol	A	SL	2.65	1.67	2.33	0.80	2	55
Luvisol	A	LS	2.65	1.59	2.45	0.70	3	56
	E	LS	2.65	1.77	2.56	0.29	4	57
Luvisol	A	SL	2.65	1.30	2.21	1.57	2	72
	A	SL	2.65	1.54	2.39	1.05	3	73
Luvisol	A	SiL	2.65	1.34	2.51	1.28	3	74
	AE	SiL	2.65	1.55	2.40	0.70	2	75
	B	SiL	2.65	1.68	2.53	0.14	3	76
Luvisol	A	SiL	2.00	1.21	1.69	2.14	3	77
	AE	SiL	2.65	1.29	2.37	0.60	3	78
	B	SiL	2.65	1.49	2.42	0.19	3	79
Luvisol	A	SL	2.00	1.39	1.66	1.50	3	80
	AE	LS	2.65	1.74	2.57	0.51	4	81
Phaeozem	A	SiL	2.00	0.83	1.73	2.33	2	19
	A	SiL	2.65	1.30	2.23	0.99	1	20
	A	SiL	2.65	1.31	2.35	0.56	2	21
	B	SiL	2.65	1.26	2.31	0.44	3	22
	C	SiL	2.65	1.42	2.40	0.13	1	23
Phaeozem	A	SiL	2.65	1.32	2.33	1.30	3	35
	A	SiL	2.65	1.44	2.28	0.76	1	36
	B	SiL	2.65	1.21	2.27	0.41	2	37
	B	SiL	2.65	1.30	2.47	0.47	3	38
	C	SiL	2.65	1.45	2.53	0.19	3	39
Phaeozem	A	SiL	2.65	1.28	2.48	1.51	3	58
	A	SiL	2.65	1.33	2.39	1.51	3	59
	A	SiL	2.65	1.30	2.38	0.89	3	60
Phaeozem	A	SiL	2.65	1.16	2.30	2.12	3	82
	A	SiL	2.65	1.38	2.59	0.99	3	83
	A	SiL	2.65	1.37	2.42	0.65	2	84

* S—sand, SL—sandy loam, SiL—silt loam, LS—loamy sand, *PD*—particle density, *BD*—bulk density measured by core method, *BDim*—bulk density by image analysis, *TOC*—total organic carbon content, *MP5*—class of number of macropore cross-sections of diameter ≥5 mm.
